# Increase in Adult Patients with Varicella Zoster Virus–Related Central Nervous System Infections, Japan

**DOI:** 10.3201/eid3012.240538

**Published:** 2024-12

**Authors:** Ayami Yoshikane, Hiroki Miura, Sayuri Shima, Masaaki Matsunaga, Soichiro Ishimaru, Yuki Higashimoto, Yoshiki Kawamura, Kei Kozawa, Akiko Yoshikawa, Akihiro Ueda, Atsuhiko Ota, Hirohisa Watanabe, Tatsuro Mutoh, Tetsushi Yoshikawa

**Affiliations:** Fujita Health University School of Medicine, Toyoake, Japan (A. Yoshikane, H. Miura, S. Shima, M. Matsunaga, Y. Higashimoto, Y. Kawamura, K. Kozawa, A. Yoshikawa, A. Ueda, A. Ota, H. Watanabe, T. Mutoh, T. Yoshikawa); Kariya Toyota General Hospital, Kariya, Aichi, Japan (S. Ishimaru)

**Keywords:** viruses, central nervous system infection, varicella zoster virus, VZV, real-time PCR, Japan, meningitis/encephalitis

## Abstract

An increase in the number of herpes zoster patients has been reported since universal varicella immunization was introduced, perhaps because of reduced opportunities for varicella patients to experience the natural booster effect caused by reexposure. We investigated recent trends of varicella zoster virus (VZV)–related central nervous system (CNS) infections at a university hospital in Japan. We enrolled patients with suspected CNS infection during 2013–2022 and tested cerebrospinal fluid samples by real-time PCR for DNA from 7 human herpesviruses. VZV DNA was the most commonly detected in 62 (10.2%) of 615 patients. Kulldorff’s circular spatial scan statistics demonstrated a significant temporal cluster of patients with VZV-related CNS infections during 2019–2022 (p = 0.008). Among persons with such infections, the percentage with aseptic meningitis was significantly higher during 2019–2022 (86.8%), when the temporal cluster of cases occurred, than during 2013–2018 (50.0%) (p = 0.0029).

Primary varicella zoster virus (VZV) infection can cause varicella (chicken pox), which is generally a mild, self-limiting disease; however, VZV infection can also rarely lead to serious complications, such as secondary bacterial superinfection of the skin, pneumonia, encephalitis, and acute cerebellar ataxia. After primary viral infection, VZV establishes latency in sensory neurons and can subsequently reactivate and cause herpes zoster infection (shingles) in elderly and immunosuppressed patients. Because of the major disease burden of varicella, the live attenuated varicella vaccine was developed in 1974 (*1*). This vaccine has been used worldwide for routine childhood immunization, and its high efficacy and safety have been demonstrated (*2*,*3*). Although the varicella vaccine was developed by researchers in Japan (*1*), for many years it was used as a voluntary base vaccine in Japan, not as a universal vaccine. However, beginning in 2014, two doses of the varicella vaccine were included in the national immunization program in Japan. Consistent with epidemiologic changes observed in other countries that have implemented universal varicella vaccination (*2*), our previous studies showed high efficacy of 2 vaccine doses for preventing VZV infection (*4*,*5*), as well as a substantial reduction in the number of varicella cases during 2015–2019, after universal immunization was implemented in 2014 (*3*).

The decrease in the number of varicella patients since universal immunization was implemented (*6*–*8*) has limited the opportunity to induce a natural booster effect in VZV-seropositive persons. The loss of this natural booster effect, which plays a role in preventing viral reactivation (*6*), accelerates the decline in immunity, leading to an increase in the number of herpes zoster infections (*7*). In fact, such increases have been reported in many countries that have initiated universal varicella vaccination (*9*–*11*), including Japan (*8*). Furthermore, the risk for herpes zoster infection has been suggested to increase with COVID-19 infection (*4*), as well as with COVID-19 mRNA vaccination (*5*). Therefore, the number of herpes zoster infections might have further increased since the onset of the COVID-19 pandemic.

In addition to herpes zoster, VZV reactivation can cause various types of central nervous system (CNS) complications, such as meningitis, meningoencephalitis, myelitis, and cerebral stroke (*12*). Several cohort studies have suggested that along with the herpes simplex virus, VZV has conferred a substantial disease burden in adult patients with CNS infections (*13*). In addition, 1 study demonstrated that the detection of VZV DNA in cerebrospinal fluid (CSF) increased the risk for subsequent dementia and epilepsy (*14*). In our recent cohort study examining the epidemiology of human herpesviruses in adult patients suspected of having CNS infections, ≈10% of those patients were positive for these viruses in the CSF; VZV was the most common (*15*). That study began in 2013; as previously mentioned, because the number of herpes zoster infections has increased over the years, the number of patients with VZV-related CNS infections might have risen as well. In this study, we sought to analyze the number of patients with VZV-related CNS disease in an adult CNS infection cohort in Japan.

## Materials and Methods

### Patient and Sample Collection

During January 2013–December 2022, we enrolled patients >15 years of age who were suspected of having CNS infection and from whom CSF was collected in the Department of Neurology of Fujita Health University School of Medicine (Toyoake, Japan). CSF was collected at time of hospital admission, and bacterial CNS infection was ruled out by negative CSF culture. CSF samples were stored at −30°C until examination. This study was approved by the Ethical Review Board of Human Studies at Fujita Health University (accession no. 14-096). Patient consent to participate in this study was obtained through an opt-out method.

### Patient Background and Clinical Characteristics

We collected patient background and clinical characteristics (specifically, sex, age, underlying conditions, diagnosis, symptoms, laboratory data, findings of brain magnetic resonance imaging [MRI] and electroencephalography, treatment, and prognosis) retrospectively from medical records. The final diagnosis for each patient was determined by the attending neurologist on the basis of clinical symptoms. Among patients with CSF pleocytosis, patients with impaired consciousness were defined as having encephalitis, whereas patients with clear consciousness were defined as having meningitis. We carried out virologic analysis, as detailed in the next sections. In VZV DNA–positive patients, we elicited herpes zoster and COVID-19 vaccination status and recent COVID-19 history through telephone interviews.

### DNA Extraction and Quantitative PCR

We extracted DNA from 200 μL of CSF using the QIAamp Blood Kit (QIAGEN, https://www.qiagen.com), eluted in 50 μL of elution buffer, then stored at −30°C before assay. We conducted real-time PCR to detect DNA of 7 human herpesviruses: herpes simplex virus (HSV) 1, HSV-2, VZV, cytomegalovirus, Epstein-Barr virus, human herpesvirus (HHV) 6, and HHV-7. The details of those real-time PCR methods for measuring viral DNA loads were described previously (*9*,*10*,*16*). The detection limit of the assays was 10 copies/tube.

### Differentiation between Oka VZV Vaccine and Wild-Type Strains

We performed differentiation between the Oka varicella vaccine (BIKEN, https://www.biken.or.jp) and wild-type strains by using a VZV loop-mediated isothermal amplification assay (LAMP) using DNA extracted from VZV-positive CSF samples. To amplify the target sequences, including 2 different single-nucleotide polymorphisms (nucleotides 105,705 and 106,262) located in the ORF62 gene, we designed primers from published sequences (GenBank accession no. NC_001348) using Primer Explorer version 3 software (https://primerexplorer.jp). We used *Sma*I to digest the LAMP products, then subjected them to electrophoresis on 1.5% agarose gels and visualized them under ultraviolet light after ethidium bromide staining (*17*).

### Statistical Analysis

We examined data pertaining to proportions, such as comparisons of patient background and clinical characteristic information, by Fisher exact or χ^2^ test. We assessed statistical comparison of numerical differences, such as laboratory findings, by using the Mann-Whitney U test. All reported p values are 2-sided. We used JMP version 12.2 (SAS Institute, https://www.sas.com) for analyses. We used Kulldorff’s retrospective space-time scan statistics, calculated using software FleXScan version 3.1 (https://sites.google.com/site/flexscansoftware), to identify temporal clusters of VZV-related CNS infections. We defined statistical significance as p<0.05.

## Results

**Figure 1:**
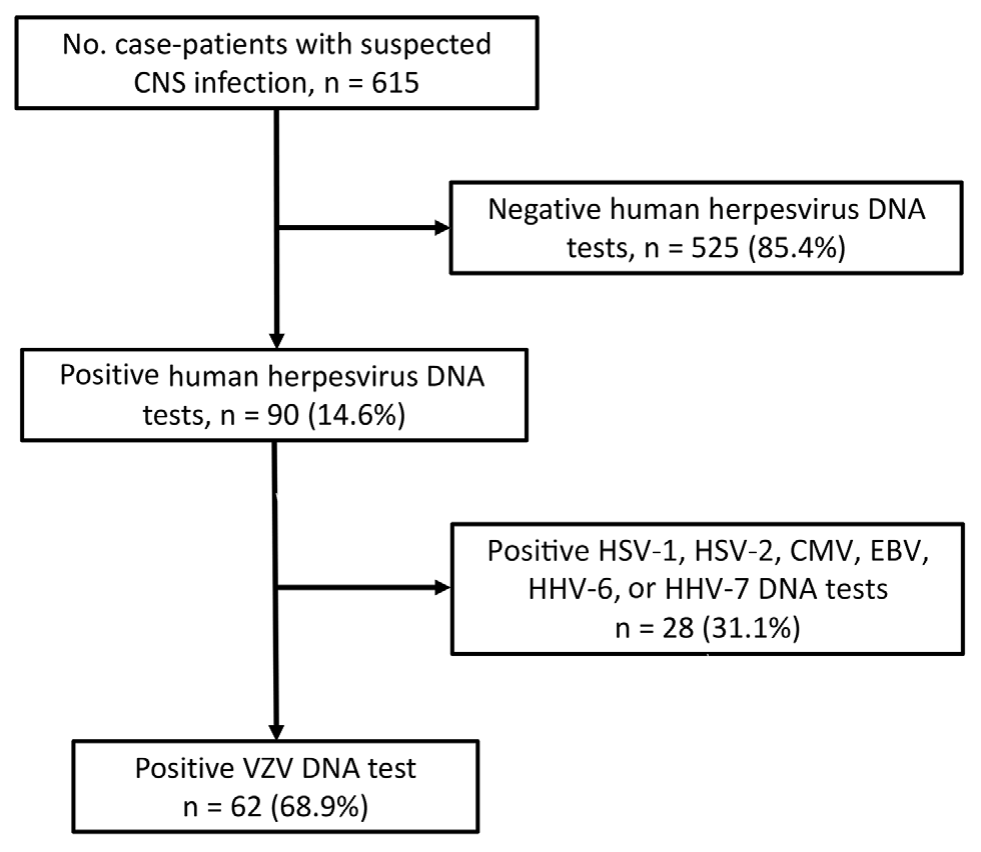
Flowchart of virologic examinations of cerebrospinal fluid samples from 615 patients with suspected CNS infection increase in adult patients with VZV-related CNS infections, Japan. Real-time PCR was carried out to detect DNA of 7 human herpesviruses: HSV-1, HSV-2, VZV, CMV, EBV, HHV-6, and HHV-7. Statistical analyses of VZV DNA–positive patients were performed to determine the trends and clinical features of VZV-related CNS infections. CMV, cytomegalovirus; CNS, central nervous system; EBV, Epstein‐Barr virus; HHV, human herpesvirus; HSV, herpes simplex virus; VZV, varicella-zoster virus.

During the observation period, a total of 615 patients (median age 53 years, range 15–91 years) were enrolled in this study. Herpesvirus DNA was detected in 90 (14.6%) of the 615 patients (Figure 1). The median age of herpesvirus DNA–positive patients was 67 years (interquartile range [IQR] 43–78 years). The most frequently detected herpesvirus was VZV (62 patients), followed by HHV-6 (10 patients), Epstein-Barr virus (10 patients), HSV-1 (7 patients), and HSV-2 (6 patients). Cytomegalovirus and HHV-7 were not detected. The median age of patients who tested positive for VZV DNA in CSF was 70.5 years (IQR 48.5–78 years); 54.8% were male and 45.2% female (Table). In total, meningitis was diagnosed in 45 (72.6%) patients, and encephalitis was diagnosed in 7 (11.3%) patients (Table). Moreover, 49 patients (79.0%) had clinical signs of herpes zoster infection, which most commonly affected the trigeminal nerve area (38.8%). Target sequences in 44 of 62 CSF samples were successfully amplified by LAMP, and all were wild-type strains. The remaining 18 samples could not be analyzed by LAMP, probably because of low copy numbers of VZV DNA.

**Table:** Comparison of background and clinical characteristics of VZV-positive patients during 2013–2018 and 2019–2022 in study of increase in adult patients with VZV-related central nervous system infections, Japan*

Characteristic	Total, N = 62	2013–2018, n = 24	2019–2022, n = 38	p value
Sex				
M	34 (54.8)	13 (54.2)	21 (55.3)	1.0000
F	28 (45.2)	11 (45.8)	17 (44.7)	
Median age (IQR)	70.5 (48.5–78)	69.5 (46.5–78.5)	70.5 (50.3–77.5)	0.9079
Underlying conditions				
Diabetes	13 (21.0)	7 (29.2)	6 (15.8)	0.2217
Hypertension	12 (19.0)	6 (25.0)	6 (15.8)	0.5111
Solid tumor/hematological malignancy	12 (19.0)	5 (20.8)	7 (18.4)	1.0000
Others	48 (77.4)	18 (75.0)	30 (78.9)	0.7617
None	11 (17.7)	4 (16.7)	7 (18.4)	1.0000
Prednisolone treatment	11 (17.7)	2 (8.3)	9 (23.7)	0.1776
Final diagnosis				
Meningitis	45 (72.6)	12 (50.0)	33 (86.8)	0.0029
Encephalitis	7 (11.3)	6 (25.0)	1 (2.6)	0.0111
Myelitis	3 (4.8)	1 (4.2)	2 (5.3)	1.0000
Hunt syndrome	4 (6.5)	2 (8.3)	2 (5.3)	0.6371
Herpes zoster†	2 (3.2)	2 (8.3)	0	0.1460
Peripheral neuritis	1 (1.6)	1 (4.2)	0	0.3871
Clinical symptoms				
Herpes zoster	49 (79.0)	22 (91.7)	27 (71.1)	0.0623
Cervical nerve	11/49 (22.4)	7/22 (31.8)	4/27 (14.8)	0.1854
Trigeminal nerve	19/49 (38.8)	9/22 (40.9)	10/27 (37.0)	1.0000
Thoracic nerve	13/49 (26.5)	6/22 (27.3)	7/27 (25.9)	1.0000
Lumbar nerve	10/49 (20.4)	3/22 (13.6)	7/27 (25.9)	0.4778
Sacral nerve	2/49 (4.1)	0/22 (0.0)	2/27 (7.4)	0.4949
Postherpetic neuralgia	23 (37.1)	10 (41.7)	13 (34.2)	0.7818
Confusion	14 (22.6)	8 (33.3)	6 (15.8)	0.1287
Laboratory data				
Pleocytosis	60 (96.8)	22 (91.7)	38 (100.0)	0.1460
Median VZV DNA copy numbers (IQR)	4.7 × 10^5^ (5.7 × 10^4^–2.3 × 10^6^)	8.2 × 10^5^ (2.8 × 10^5 ^–5.7 × 10^6^)	4.2 × 10^5^ (5.3 × 10^4^–1.0 × 10^6^)	0.1255
Imaging data				
Abnormal brain MRI finding	9/50‡ (18.0)	7/21 (33.3)	2/29 (6.9)	0.0253
Abnormal EEG finding	7/20§ (35.0)	5/14 (35.7)	2/6 (33.3)	1.0000
Treatment				
Intravenous acyclovir administration	61 (98.4)	24 (100.0)	37 (97.4)	1.0000
Median duration, d (IQR)	14 (10–15)	14 (11.8–15.5)	14 (10–14.6)	0.6480
Adjunctive prednisone	10 (16.1)	2 (8.3)	8 (21.1)	0.0750
Prognosis				
Sequelae	33 (53.2)	14 (50.0)	19 (50.0)	0.4398
Fatal	1 (1.6)	1 (4.2)	0	0.3871

*Values are no. (%) except as indicated. EEG, electroencephalogram; IQR, interquartile range; MRI, magnetic resonance imaging; VZV, varicella  zoster virus. †The classification of herpes zoster as the final diagnosis indicates that those patients were not categorized under any of the other diseases listed in this table; they are classified as having herpes zoster only. ‡Of 62 patients with VZV-related central nervous system infections, 50 underwent brain MRI. §Of 62 patients with VZV-related central nervous system infections, 20 underwent EEG.

In Japan, the live-attenuated varicella zoster Oka vaccine (BIKEN) was additionally approved in March 2016, and the recombinant subunit vaccine Shingrix (https://www.shingrix.com) was approved in March 2018 as zoster vaccine for adults >50 years of age. Of 62 patients who had VZV-related CNS infections develop after 2016, a total of 49 were eligible for zoster vaccination before those CNS infections developed. However, among the 26 patients for whom vaccination status was available, none had received the zoster vaccine. Furthermore, to investigate the relationship between an increase in VZV-related CNS infections and COVID-19 illness or COVID-19 vaccine, we elicited COVID-19 history and COVID-19 vaccination status from patients who had VZV-related CNS infection after 2020. Of the 19 patients for whom COVID-19 history was available, none had COVID-19 before the onset of CNS infection, whereas VZV-related CNS infections developed in 6 patients after they received COVID-19 vaccinations.

**Figure 2:**
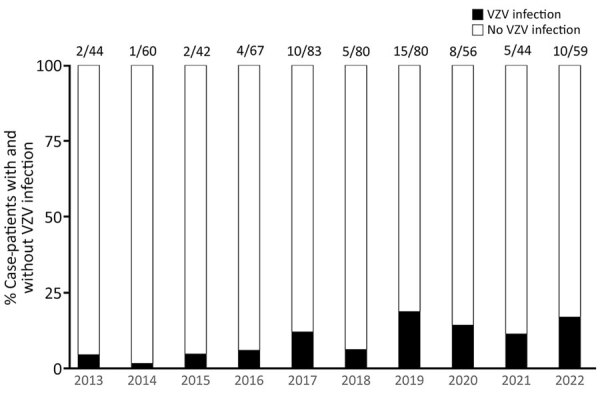
Trends of VZV-related central nervous system infections at Fujita Health University School of Medicine during 2013–2022 in study of increase in adult patients with VZV-related central nervous system infections, Japan. Vertical bars show percentages of patients with (black) and without (white) VZV infection each year. Numbers above bars indicate no. patients positive/no. analyzed. VZV, varicella-zoster virus.

The proportion of VZV DNA–positive patients among patients suspected of having CNS infection appeared to be increasing (Figure 2). Kulldorff’s circular spatial scan statistics demonstrated a significant temporal cluster of patients with VZV-related CNS infections during 2019–2022 (p = 0.008). This time frame was defined as the late period, in contrast with the early period of 2013–2018. However, the disease trends among hospitalized patients in our institution did not change during the overall study period (2013–2022) (Appendix Figure).

Next, we compared patients’ background and clinical characteristics between the early and late periods (2013–2018 vs. 2019–2022) (Table). Background factors (sex, median age, number of underlying conditions, and number receiving prednisolone treatment) did not differ significantly between the 2 periods. However, the percentage of patients with several VZV-related CNS infections differed significantly between the 2 periods. The percentage of patients with aseptic meningitis was significantly higher in the late period (33/38 cases, 86.8%) than in the early period (12/24 cases, 50.0%; p = 0.0029). Meanwhile, the percentage of patients with encephalitis was significantly higher in the early period (6/24 cases, 25.0%) than in the late period (1/38, 2.6%; p = 0.0111). The percentage of patients with herpes zoster infection was higher in the early period (22/24 cases, 91.7%) than in the late period (27/38 cases, 71.1%). We observed no significant difference between the 2 periods either in the percentage of patients with pleocytosis or in the VZV viral load in CSF. The percentage of patients with abnormal brain MRI findings was significantly higher in the early period than in the late period (33.3% vs. 6.9%; p = 0.0253).

## Discussion

This cohort study, initiated in 2013, showed that, beginning in 2019, the number of patients with VZV-related CNS infections increased significantly among patients at our institution in Japan. Furthermore, Kulldorff’s circular spatial scan statistics, which are used to elucidate clusters of infectious diseases, including COVID-19, demonstrated a statistically significant temporal cluster of patients with VZV-related CNS infections during 2019–2022 (*18*,*19*). Bryant et al. (*11*) recently performed a molecular epidemiologic study on VZV on the basis of samples collected from patients with CNS infection in New York, USA, and showed a similar increase in patients with VZV-related CNS infection. However, because those study samples were transported to a central laboratory from many hospitals across New York state, whether this trend represents a real increase in disease incidence or an increase in the submission of samples for testing is unclear. Our results of a single-center study clearly demonstrated a recent increase in the number of VZV-related CNS infections, a pattern similar to that recently observed regarding the incidence of herpes zoster infection (*20*), which strongly supports the findings demonstrated by Bryant et al. (*11*). On the basis of the Hope-Simpson model, it has been suggested that the greater number of herpes zoster patients may be caused in large part to reduced opportunities for persons previously infected with varicella to experience the natural booster effect caused by reexposure (*6*). Although the increased incidence of herpes zoster has been demonstrated in many industrial countries with aging populations (*20*), no clear correlation has been demonstrated to date between this increase and the lessened opportunity for the natural booster effect (*21*). The higher incidence of herpes zoster is thought to be associated with increasingly aging populations (*22*) and the growing number of patients at high risk for herpes zoster (*23*), such as immunocompromised persons or persons with diabetes or autoimmune diseases. In any event, data suggest that herpes zoster infection and VZV-related CNS infections should be monitored to accurately assess the disease burden associated with VZV reactivation in adults, especially in aging populations.

Some have suggested that the risk for herpes zoster is increased by COVID-19 (*4*) and COVID-19 mRNA vaccination (*5*), and some case reports have indicated that zoster meningitis developed after COVID-19 mRNA vaccination (*24*–*26*). Among the patients in our study with an available history of COVID-19 infection and vaccination status, none had COVID-19 before the onset of the VZV-related CNS infection, but VZV-related CNS infections developed in 6 patients after they had COVID-19 vaccinations. Our study is insufficient to elucidate an association between VZV-related CNS infection and COVID-19 or COVID-19 vaccination; further studies are needed to clarify this issue.

Postherpetic neuralgia has been considered to be the greatest contributor to disease burden caused by VZV reactivation, and it has been demonstrated that both live-attenuated and subunit zoster vaccines reduced the risks of herpes zoster infection and postherpetic neuralgia (*27*). This study shows that in addition to herpes zoster and postherpetic neuralgia, VZV-related CNS infections might be a cause of the disease burden associated with VZV reactivation. The zoster vaccine is expected to reduce the risk for VZV-related CNS infections, as has already been shown for herpes zoster and postherpetic neuralgia (*27*). Recent studies have suggested that VZV reactivation might be associated with the pathogenesis of more severe CNS diseases, such as brain infarction (*28*) and dementia (*29*). Furthermore, large cohort analyses have demonstrated that the zoster vaccine can reduce the risk for brain infarction (*17*) and dementia (*18*). In this study, no patients with VZV-related CNS infection for whom vaccination history was available had received the zoster vaccine. Therefore, determining whether the zoster vaccine can reduce the burden of additional diseases, including VZV-related CNS infection, is key. Such studies will provide information to aid in determining the ability of the zoster vaccine to reduce healthcare costs in aging populations and in evaluating the cost-effectiveness of implementing universal zoster vaccination.

To determine whether the clinical features of VZV-related CNS infections differed at our institution before and after the beginning of the temporal cluster of those diseases in 2019, we compared patients’ background and clinical characteristics during 2013–2018 with those during 2019–2022. Of note, most patients (86.8%) in the late period received a diagnosis of aseptic meningitis, compared with only half in the early period (p = 0.0029). Conversely, incidence of encephalitis was significantly higher in the early period (p = 0.014). In addition, a significantly higher percentage of patients had abnormal brain MRI findings in the early period (p = 0.0253), suggesting a high frequency of encephalitis patients in that period. Although a previous study found that VZV meningitis patients were significantly younger than VZV encephalitis patients (*19*), no statistically significant difference in age was observed between the 2 periods in this study. We did not change the criteria for performing a spinal tap or brain MRI during the study; further research is needed to clarify the reasons for the recent increase in the number of patients with VZV-related aseptic meningitis. Furthermore, most patients with VZV-related CNS infections before 2018 had herpes zoster infection, whereas in the late period, ≈30% of the patients did not have a zosteriform rash (zoster sine herpete); however, no significant difference was observed in frequency. We recently demonstrated that the reactivated Oka vaccine strain caused aseptic meningitis in a child without herpes zoster virus (*30*). Although some physicians might previously not have measured VZV DNA in CSF collected from patients without the typical herpes zoster rash, the recent introduction of comprehensive PCR panel tests, such as FilmArray (bioMérieux, https://www.biomerieux.com), might reveal the precise incidences of VZV-related CNS infections that occur without this rash.

Molecular epidemiologic analysis in this study demonstrated that all evaluated VZV DNA in CSF was derived from wild-type VZV. In Japan, 2 doses of varicella vaccination were introduced as part of the national immunization schedule in 2014. Meanwhile, 2 different zoster vaccines, specifically Shingrix (a recombinant subunit vaccine) and varicella-zoster Oka vaccine (a live-attenuated vaccine), have been licensed and are available in Japan, whereas only Shingrix is currently recommended for herpes zoster vaccination in the United States (*31*). Therefore, some elderly persons have received a live-attenuated zoster Oka vaccine, and the number of recipients of that vaccine is expected to increase dramatically in the future. Although the risk of viral reactivation of the Oka vaccine strain was shown to be lower than that of wild-type VZV on the basis of in vivo (*32*) and in vitro (*33*) studies, the Oka vaccine strain is well known to be capable of reactivating and causing herpes zoster infection (*34*). In addition, a recent study detected Oka vaccine strain DNA in CSF collected from patients with CNS infection, although the number of patients was very small, and all were children and young adults (*11*). Therefore, the reactivated Oka vaccine strain could feasibly cause VZV-related CNS infections, and molecular epidemiologic analysis to distinguish between wild-type and vaccine-type strains will become increasingly key in future.

## Conclusions

In this study, we investigated 10-year trends and clinical features of VZV-related CNS infections in adult patients with suspected CNS infection at our university hospital in Japan. Statistical analysis revealed a significant temporal cluster of patients with VZV-associated CNS infections during 2019–2022, as well as an increasing proportion of aseptic meningitis caused by VZV reactivation during that period. Although all detected VZV DNA was the wild-type strain in this study, molecular epidemiologic studies to differentiate between vaccine and wild-type strains will be key in the future.

Appendix Additional information about increase in adult patients with varicella-zoster virus–related central nervous system infections, Japan
