# Supplementary material for: Increase in Adult Patients with Varicella Zoster Virus–Related Central Nervous System Infections, Japan
Source: Emerg Infect Dis. 2024 Dec;30(12):2476–82. doi: 10.3201/eid3012.240538 (PMC11616634; doi:10.3201/eid3012.240538)
Supplement: Appendix — Additional information about increase in adult patients with varicella-zoster virus–related central nervous system infections, Japan [file 24-0538-Techapp-s1.pdf]

Article DOI: https://doi.org/10.3201/eid3012.240538

Increase in Adult Patients with Varicella Zoster Virus–Related Central Nervous System Infections, Japan

Appendix

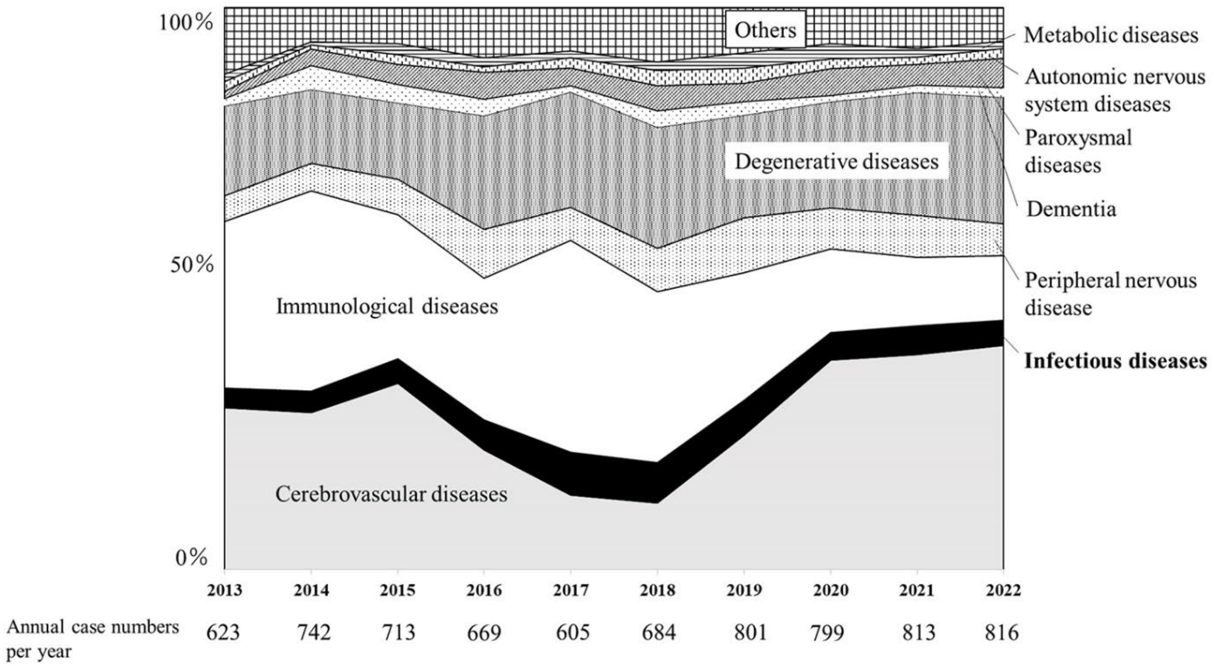

Appendix Figure. Groups of diseases experienced by patients hospitalized in the Department of Neurology of our university hospital from 2013 to 2022. The chart shows the percentage of patients with each disease group on a yearly basis.
